# Adipose Extracellular Matrix/Stromal Vascular Fraction Gel Secretes Angiogenic Factors and Enhances Skin Wound Healing in a Murine Model

**DOI:** 10.1155/2017/3105780

**Published:** 2017-08-01

**Authors:** Mingliang Sun, Yunfan He, Tao Zhou, Pan Zhang, Jianhua Gao, Feng Lu

**Affiliations:** Department of Plastic and Cosmetic Surgery, Nanfang Hospital, Southern Medical University, 1838 Guangzhou North Road, Guangzhou, Guangdong 510515, China

## Abstract

Mesenchymal stem cells are an attractive cell type for cytotherapy in wound healing. The authors recently developed a novel, adipose-tissue-derived, injectable extracellular matrix/stromal vascular fraction gel (ECM/SVF-gel) for stem cell therapy. This study was designed to assess the therapeutic effects of ECM/SVF-gel on wound healing and potential mechanisms. ECM/SVF-gel was prepared for use in nude mouse excisional wound healing model. An SVF cell suspension and phosphate-buffered saline injection served as the control. The expression levels of vascular endothelial growth factor (VEGF), basic fibroblast growth factor (bFGF), and monocyte chemotactic protein-1 (MCP-1) in ECM/SVF-gel were analyzed at different time points. Angiogenesis (tube formation) assays of ECM/SVF-gel extracts were evaluated, and vessels density in skin was determined. The ECM/SVF-gel extract promoted tube formation in vitro and increased the expression of the angiogenic factors VEGF and bFGF compared with those in the control. The expression of the inflammatory chemoattractant MCP-1 was high in ECM/SVF-gel at the early stage and decreased sharply during the late stage of wound healing. The potent angiogenic effects exerted by ECM/SVF-gel may contribute to the improvement of wound healing, and these effects could be related to the enhanced inflammatory response in ECM/SVF-gel during the early stage of wound healing.

## 1. Introduction

Wound healing is a highly complex process that remains a major challenge in modern medicine. Among the factors contributing to these nonhealing conditions, impairment of cytokine production and reduced vascularization play crucial roles [1]. Mesenchymal stem cells- (MSCs-) based cytotherapy is an attractive approach in wound healing due to the differentiation potential, immunomodulating properties, and paracrine effects of MSCs [2–5]. Among the available MSCs, adipose-derived stem cells (ASCs) are promising candidates for cytotherapy because they can be easily harvested from adipose tissue and are abundant in number [6, 7]. However, isolation of ASCs still requires enzymatic digestion, which increases the risk of biological contamination. Additionally, in vitro culture and expansion of ASCs require specific laboratory experience and can take days to weeks. Moreover, in most studies, ASCs suspensions are injected separately without the protection of extracellular matrix (ECM) components, leading to rapid elimination of ASCs by the immune system and subsequent poor cell retention at recipient sites [8–10]. All of these factors weaken the therapeutic effects and applications of ASCs-based cytotherapy.

We have recently described a novel adipose-tissue-derived injectable extracellular matrix/stromal vascular fraction gel (ECM/SVF-gel) [11]. Taking advantage of shearing force (mechanical emulsification of adipose tissue by shifting between two 10 mL syringes connected by a female-to-female Luer-Lok connector) to selectively break mature adipocytes, the ECM/SVF-gel eliminates most of the lipids and other unwanted components (such as tumescent fluid) of Coleman fat. The shear force causes more damage to adipocytes than to stromal vascular fraction (SVF) cells components due to the large size and fragility of the cells, leaving only the extracellular matrix (ECM) and SVF cells and greatly improving ASCs density. According to our previous results, standard Coleman fat mechanically processed for 1 min in a 10 mL syringe at a flow rate of 10 mL/s will lead to maximum enrichment of viable ASCs and minimal ECM fragmentation. This procedure is considered optimal for producing ECM/SVF-gel.

Because ECM/SVF-gel is rich in ASCs, we speculated that ECM/SVF-gel may exert cytotherapeutic effects on wound healing. To test this hypothesis, preliminary studies were conducted in a mouse excisional wound healing model to assess the therapeutic value of ECM/SVF-gel for wound repair. First, angiogenic factors expression and angiogenic effect of ECM/SVF-gel extract in vitro were tested. Next, angiogenic factors secretion and angiogenic efficacy of the grafted ECM/SVF-gel in vivo were examined. Finally, the inflammation level of ECM/SVF-gel in vivo was evaluated.

## 2. Materials and Methods

### 2.1. Fat Harvesting and ECM/SVF-gel Preparation

**Figure 1:**
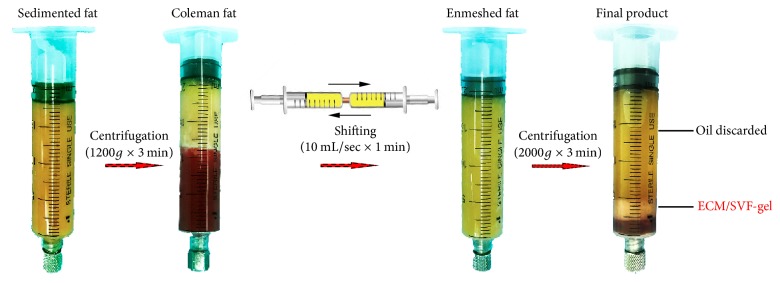
The fabrication of ECM/SVF-gel.

Human abdominal lipoaspirates were obtained from female abdominal lipoaspirates after informed consent and Nanfang Hospital ethics committee approval. ECM/SVF-gel was prepared as per the manufacturer's instruction [11]. Briefly, liposuction was performed with a 3 mm multiport cannula and the harvested fat was allowed to stand for 10 min in ice water. The liquid portion was then discarded and the fat layer was then centrifuged at 1200*g* for 3 min to generate Coleman fat. After centrifugation and discarding the liquid portion, the obtained Coleman fat was then mechanically emulsified by shifting between two 10 mL syringes connected by a female-to-female Luer-Lok connector with an internal diameter of 2.4 mm for 1 min (10 mL/sec). After processing, the fat turned into an emulsion and was then processed by centrifugation at 2000*g* for 3 min. Finally, the remaining substance under the oil layer was ECM/SVF-gel (Figure 1). For ASCs extraction, the ECM/SVF-gel was digested with 0.075% collagenase for 30 min on a shaker at 37°C. After centrifugation at 800*g* for 5 min, the cell pellets from ECM/SVF-gel were then filtered through a 100 *μ*m mesh and SVF cells' number was then counted. The remaining SVF cells were cultured until 80% confluence. Passage 3 to Passage 5 ASCs were used in the following experiments.

### 2.2. Scanning Electron Microscopy (SEM)

The surface characteristics of ECM/SVF-gel were determined by scanning electron microscopy (SEM) (Hitachi S-3000N; Hitachi Ltd., Tokyo, Japan). Samples were mounted on a carbon tape and coated with gold prior to imaging. An acceleration voltage of 20 kV was used and images were observed using a Canon digital camera (Canon Inc., Tokyo, Japan).

### 2.3. Multilineage Differentiation Assay

Assays of adipogenic, osteogenic, and chondrogenic lineages were conducted in a control medium supplemented with one of the three formulae as previously described [12]. Cultured ASCs were stained with Oil-Red O, Alizarin Red S, and Alcian Blue to identify fat, bone, and cartilage cells, respectively.

### 2.4. Healing of Full-Thickness Wounds in Nude Mice

Female nude mice (4–6-week-old) were purchased from the Southern Medical University Laboratory Animal Center and maintained in microisolator cages at the Animal Experiment Center of Nanfang Hospital. All animal experiments were approved by Nanfang Hospital Institutional Animal Care and Use Committee and conducted according to the guidelines of the National Health and Medical Research Council (China). After general anesthesia, the mice were depilated and two 6 mm radius full-thickness excisional wounds were produced on the back of every mouse. A silicone ring was secured around the wound to prevent wound contraction. 54 mice were divided into ECM/SVF-gel treated group (injection of ECM/SVF-gel into the four quadrants, *n* = 18), SVF cell treated group (*n* = 18), and control group (injection of PBS, *n* = 18). A local injection of ECM/SVF-gel or an SVF cell suspension contains the same number of SVF cells (2 × 10^4^). After treatment, a Tegaderm sterile dressing (3M Healthcare, St. Paul, MN) was used to cover the wounds and was replaced every other day. The wound area was photographed with a digital camera and quantified using ImageJ software (NIH, Bethesda, MD).

### 2.5. Histological Examination and Capillary Density Measuring

Skin and ECM/SVF-gel around the wounds were harvested at days 2, 4, 7, 10, and 14. One half of the samples were paraffin-embedded for hematoxylin and eosin (HE) staining and the rest were snap-frozen and preserved at −80°C for RNA analysis. For HE staining, paraffin-embedded samples were cut into 4 *μ*m sections and stained with hematoxylin for 5 min and washed with Scott's solution three times.. Samples were treated with 1% HCl for 20 s and then rinsed with Scott's solution for 1 min. After incubation in 80% ethanol for 2 min, sections were stained with eosin for 2 min. Images were taken using a Nikon E200 microscope (Nikon Corp., Tokyo, Japan). Vascularization of skin was assessed by counting the capillaries in 8 fields of each HE section, performed by two blinded reviewers.

### 2.6. ECM/SVF-gel Extract Preparation

ECM/SVF-gel extract was prepared according to the manufacturer's instructions [13]. Briefly, in vivo ECM/SVF-gel samples from all 5 time points were harvested. The samples were cut into small pieces and mixed with an equal volume of Dulbecco's modified Eagle's medium. The mixture was incubated for 24 h at 37°C in a CO_2_ incubator. ECM/SVF-gel extracts were collected after the mixture was centrifuged at 12,000 rpm for 5 min and sterile-filtered through a 0.22 mm filter (Sarstedt, Nümbrecht, Germany). The extracts were stored at −20°C until use.

### 2.7. Cytokines Expression in ECM/SVF-gel Extract and Tube Formation Assay

Serum samples were collected from each mouse at all time points. Expressions of vascular endothelial growth factor (VEGF), basic fibroblast growth factor (bFGF), and monocyte chemotactic protein-1 (MCP-1) in ECM/SVF-gel extracts or serum were determined by high sensitivity enzyme-linked immunosorbent assay kits (R&D Systems). For tube formation assay, human umbilical vein endothelial cells (HUVECs) were obtained from the Research Laboratory Collaboration Alliance of Nanfang Hospital (Guangzhou, China). Culture plate and the experimental equipment were precooled at −20°C before operation; Matrigel (Becton, Dickinson & Co., 356230, Franklin Lakes, NJ) was added to the culture plate and solidified by incubation at 37°C for 2 h before seeding cells. HUVECs (2 × 10^4^ per well) mixed with 1 mL of ECM/SVF-gel extract from 7-day samples were then seeded in the culture plate. As a positive control, the HUVECs in the culture plate were treated with angiogenic growth factors (10 ng/mL vascular endothelial growth factor (VEGF; R&D Systems, MN, USA) and 1 ng/mL basic fibroblast growth factor (bFGF; Sigma)). As a control, HUVECs alone were cultured in normal culture medium. Angiogenesis (tube formation) was observed and photographed in high-powered fields (HPF) after 24 h.

### 2.8. Quantitative Real-Time Polymerase Chain Reaction (PCR)

RNA was isolated from skin and ECM/SVF-gel samples using TRIzol Reagent (Invitrogen, Life Technologies, Carlsbad, CA, USA) according to the manufacturer's protocol. Reverse transcription was performed using a cDNA synthesis kit (Thermo Scientific, USA). Primer sequence for PPAR*γ* was designed. Quantitative PCR was performed using FastStart Universal SYBR Green Master (Rox). PCR specificity was assessed by the Ct method using *β*-actin as an endogenous reference gene.

### 2.9. Statistical Analysis

Data were expressed as mean ± SD (standard deviation). Results were analyzed with one-way analyses of variance (SPSS, version 21; IBM Corporation, Armonk, NY, USA). Furthermore, an independent Student's *t*-test of two groups at a single time point was performed. A value of *P* < 0.05 was considered statistically significant.

## 3. Results

### 3.1. Structural Characteristics of ECM/SVF-gel and Fresh Adipose Tissues

**Figure 2:**
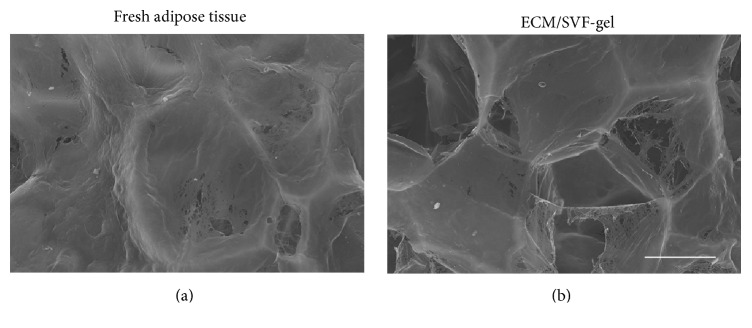
SEM analysis of ECM/SVF-gel and fresh adipose tissues. SEM analysis revealed the structural characteristics of fresh adipose tissue sample (a) and ECM/SVF-gel (b). Scale bar = 50 *μ*m.

ECM/SVF-gel and fresh adipose tissues were evaluated by SEM. The control adipose tissues had a relatively well integrated ECM structure (Figure 2(a)). The ECM integrity in ECM/SVF-gel decreased slightly after mechanical processing (Figure 2(b)).

### 3.2. Multilineage Differentiation of ASCs Isolated from ECM/SVF-gel

**Figure 3:**
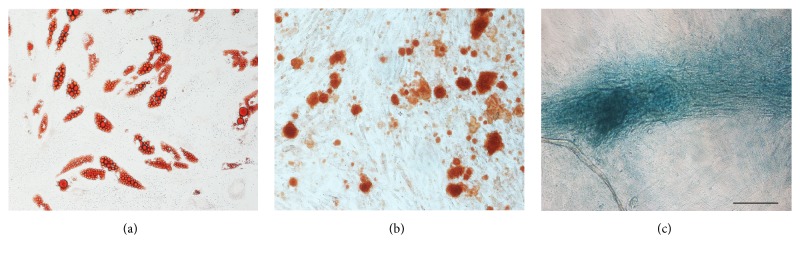
Multilineage differentiation of ASCs isolated from ECM/SVF-gel. Multilineage differentiation of ASCs isolated from ECM/SVF-gel underwent adipogenic (a), osteogenic (b), and cartilage differentiation (c). Scale bar = 200 *μ*m.

ASCs isolated from ECM/SVF-gel grew well in vitro. To verify multipotent differentiation, ASCs were incubated in a medium known to induce adipogenic, osteogenic, and chondrogenic lineages. Cells contained lipid droplets, exhibited matrix mineralization, expressed cartilage-specific proteoglycans, and were stained positively for Oil-Red O (Figure 3(a)), Alizarin Red S (Figure 3(b)), and Alcian Blue (Figure 3(c)), respectively.

### 3.3. ECM/SVF-gel Promoted Wound Healing

**Figure 4:**
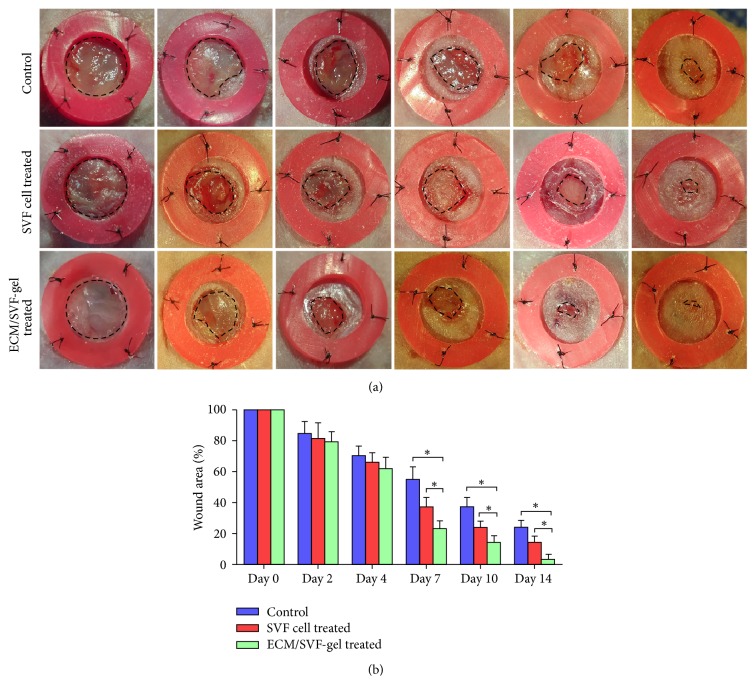
Wound healing in nude model. 6 mm radius cutaneous wounds were created on dorsal areas of nude mice and treated with ECM/SVF-gel (2 × 10^4^ cells, *n* = 18), SVF cells (2 × 10^4^ cells suspended in 0.1 mL of PBS, *n* = 18), and PBS injection (*n* = 18), respectively. (a) All injections were made subdermally into the four quadrants of the wound. (b) Quantification of wound area at different time points (^*∗*^*P* < 0.05).

The therapeutic value of ECM/SVF-gel and SVF in wound healing was evaluated in a nude mouse excisional wound healing model. Healing of wounds was significantly more rapid with ECM/SVF-gel injection compared with that of the SVF cell suspension and the PBS control on day 7. On day 14, the ECM/SVF-gel achieved complete wound healing, whereas wounds in the groups treated with SVF cell suspension and PBS remained unhealed (Figure 4).

### 3.4. ECM/SVF-gel Extracts Promoted Vascularization In Vitro

**Figure 5:**
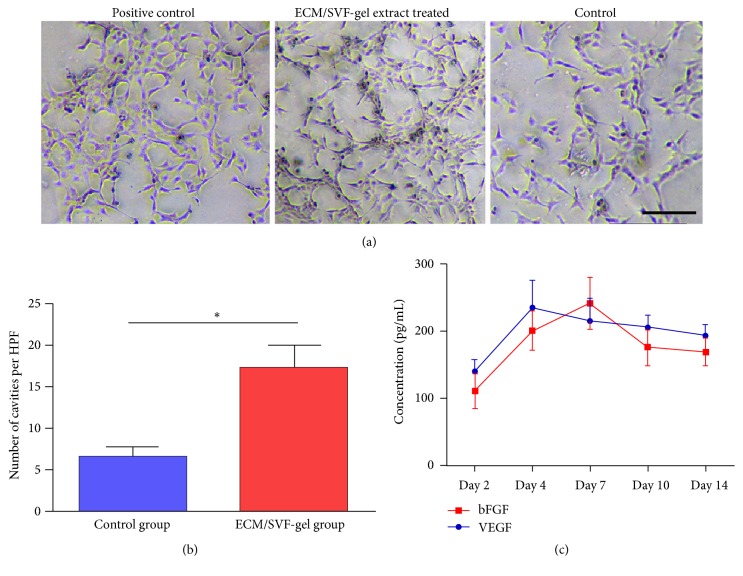
Angiogenesis assay and angiogenic factors expression. (a) HUVECs in ECM/SVF-gel extract (7-day samples) treated group and in positive control group formed numerous interconnected tubular structures at 12 hours while HUVECs did not connect to the vascular net in the control group. Scale bar = 200 *μ*m. (b) Quantitative analysis of tube formation. (c) Quantitative analysis of angiogenic factors in ECM/SVF-gel extract at different time points (^*∗*^*P* < 0.05).

In an angiogenesis (tube formation) assay, treatment with ECM/SVF-gel extracts derived from day 7 for 24 h resulted in the formation of interconnected tubular structures of HUVECs, whereas HUVECs were unable to connect to the vascular net in the control group (Figures 5(a)-5(b)). VEGF and bFGF expression levels in ECM/SVF-gel extracts at different time points were measured by enzyme-linked immunosorbent assays. The results revealed high concentrations of VEGF and bFGF (Figure 5(c)).

### 3.5. ECM/SVF-gel Enhanced Vascularization In Vivo

**Figure 6:**
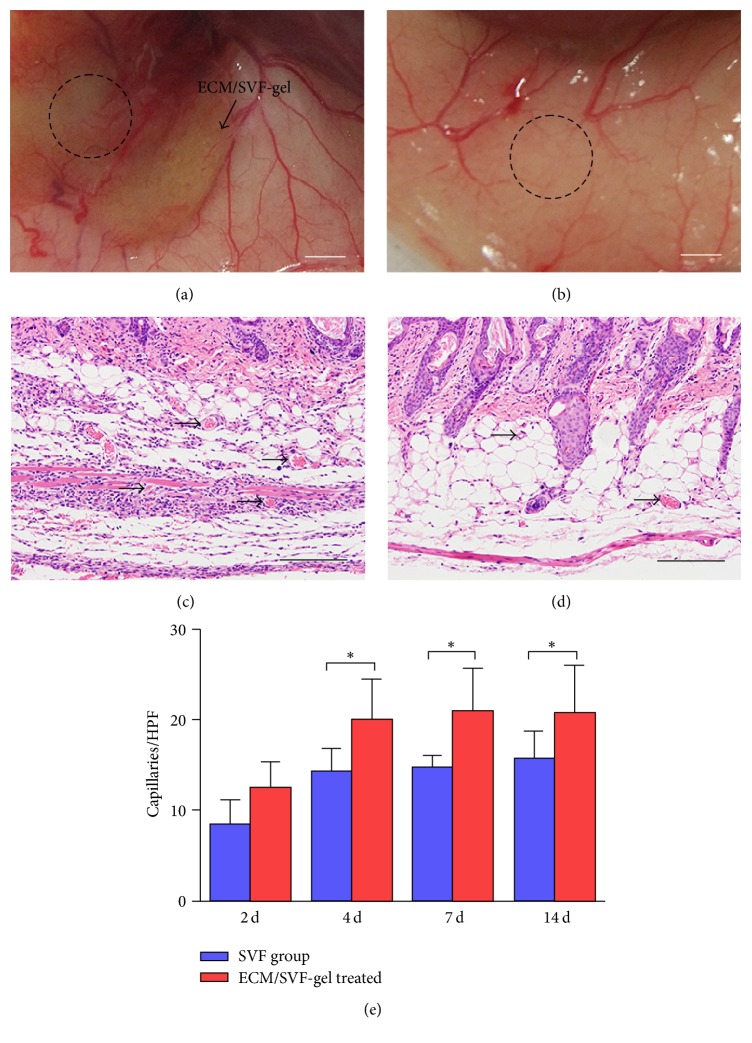
Vascularization and Immunofluorescence staining. Macroscopic visualization of subcutaneous blood vessels around the wound (dotted area) in ECM/SVF-gel treated group (a) and SVF cell treated group (b) at day 30. Scale bar = 2 mm. Observation of vessels (arrows) in skin HE stained sections in the ECM/SVF-gel treated group (c) and SVF cell treated group (d) at day 7. Scale bar = 200 *μ*m. Quantification of capillary densities in HE stained skin sections (e) (^*∗*^*P* < 0.05).

Macroscopic visualization of subcutaneous blood vessels around the wound was performed on day 30. In the ECM/SVF-gel group, around the wound, vessels and their fine branches extended into the ECM/SVF-gel, forming networks of blood vessels and nourishing the wound (Figure 6(a)). In wounds in the SVF group, vessels were limited to the skin surrounding the wounds (Figure 6(b)). Observation of vascularization in skin HE stained sections revealed that ECM/SVF-gel-induced promotion of vascularization (Figure 6(c)) was significantly greater than that in the SVF group (Figure 6(d)). Quantification of capillary densities in HE stained skin sections was then performed. Capillary density was significantly higher in the ECM/SVF-gel treated group than in the SVF treated group from day 4 to day 14 (Figure 6(e)).

**Figure 7:**
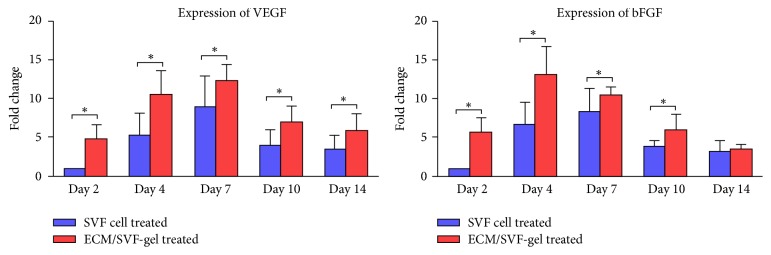
Expression of angiogenic factors in skin samples. VEGF and bFGF expression of skin samples in the ECM/SVF-gel treated group and SVF group at different time points (^*∗*^*P* < 0.05).

The RNA expression level of the angiogenic factor VEGF peaked on day 7 and was higher in the ECM/SVF-gel treated group than in the SVF treated group at all time points, whereas expression of the angiogenic factor bFGF peaked on day 4 in the ECM/SVF-gel treated group and was higher than that in the SVF treated group between days 2 and 10 (Figure 7).

### 3.6. Inflammatory Reaction in ECM/SVF-gel

**Figure 8:**
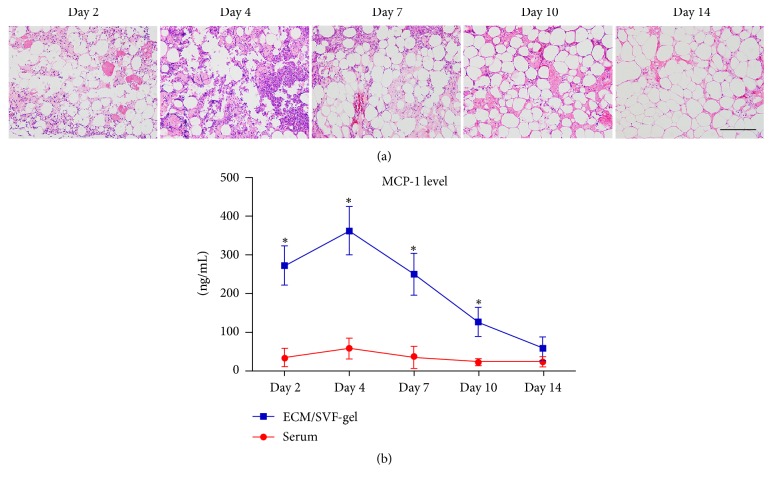
HE staining of ECM/SVF-gel and MCP-1 expression. (a) Inflammatory cell infiltration was observed obviously in ECM/SVF-gel at an early stage and declined at a late stage of wound healing. Scale bar = 200 *μ*m. (b) MCP-1 expression in ECM/SVF-gel and serum at different time points (^*∗*^*P* < 0.05).

Obvious inflammatory cell infiltration was found in ECM/SVF-gel at the early stage and declined during the late stage of wound healing (Figure 8(a)). The expression of MCP-1 from ECM/SVF-gel extracts peaked on day 4 and decreased sharply thereafter, showing higher levels than that in serum from day 2 to day 10 (Figure 8(b)).

## 4. Discussion

Adipose ECM/SVF-gel, a novel injectable consisting of adipose native ECM and functional ASCs, was applied in this study to optimize cytotherapy in wound healing. The results revealed that the ECM/SVF-gel treated group showed more rapid wound closure than did the SVF group. The enhanced expression of angiogenic factors in ECM/SVF-gel and a rich vascular network around the wound area indicated that the faster healing of wounds in the ECM/SVF-gel group could be attributed to increased vascularization. Vascularization of the wound bed is known to play a significant role in the wound healing process [13, 14]. Recent cytotherapy strategies using MSCs have aimed at increasing vascularization to achieve wound healing due to their paracrine effects and differentiating potential [15, 16]. Despite demonstrating clear therapeutic potential, limitations still remain regarding the clinical application of MSCs, primarily because of their low therapeutic efficacy and biosafety concerns [17, 18]. Moreover, in most studies of cytotherapy for cutaneous wound healing, MSCs are subdermally injected into or around the wound area. Although this strategy has been shown to promote wound healing, the ultimate therapeutic potential of MSCs appears to be limited because of poor cell retention and reduced cell potency at the wound site [19, 20]. In previous decades, studies have attempted to optimize MSC-based therapeutics either by enhancing the expression of trophic factors or through prevention of MSC death at the recipient site. Overexpression of trophic factors through transgenic methods or by exposure to potency-enhancing factors has been used successfully in animal models [21–23]. Despite the success of some transgenic strategies, the tumorigenic potential of MSCs due to insertional mutations resulting from gene transfer is still a major safety concern for clinical translation. Various synthetic materials have been developed to provide carrier scaffolds that mimic ECM properties to enhance cytotherapy [24–26]. The advantages of synthetic materials and scaffolds mainly rely on the technical possibility that chemical and physical properties (e.g., porosity and surface characteristics) can be specifically designed [27]. However, native ECM exhibits biocompatibility, biodegradability, and inherent biological functions that could make it more suitable for a range of cytotherapy applications [28, 29].

Wound healing is a dynamic and complex process involving hemostasis, inflammation, proliferation, and remodeling [30, 31]. Multiple studies have indicated that inflammatory cells are an important source of the overall proangiogenic stimulus in the healing process [32–34]. Tissue injury causes a rapid acute inflammatory response designed to clear microbes, dying cells, and debris. In response to specific chemoattractants, such as fragments of ECM protein, transforming growth factor *β*, and MCP-1, a large number of monocytes infiltrate the wound site and become activated macrophages, which produce high levels of cytokines, such as platelet-derived growth factor (PDGF), VEGF, and bFGF [35, 36]. In response to increased levels of cytokines, vascularization occurs, providing oxygen and nutrients to sustain high levels of cellular activity and promote reepithelialization of wounds. In this study, significant expression of the inflammatory chemoattractant MCP-1 and obvious inflammatory cells infiltration can be found in ECM/SVF-gel during the early stage of wound healing, and inflammation levels decrease markedly during the late stage. The intense inflammatory response of ECM/SVF-gel in the early stage is likely to contribute to the secretion of angiogenic factors and subsequent vascularization in the wound area. Actually, the implanted ECM/SVF-gel around the wound served only as a stimulus that recruited inflammatory cells from the recipient mouse. Then, the recruited cells became a source and secreted a large amount of angiogenic factors (VEGF and bFGF) that promoted the vascularization and healing of the wound. This process is much similar to the remodeling of grafted adipose tissue in vivo, during which numerous host inflammatory cells infiltrated in the graft and scavenged oil drop in necrotizing zone [37]. Moreover, the angiogenesis of the adipose tissue after transplantation mainly originated from the recipient environment and most of the regeneration cells and secreted growth factors that take part in adipogenesis under adipose tissue inflammation condition come from recipient circulation [38, 39].

Studies have suggested that persistent high levels of inflammation cause fibrosis and impair wound healing, whereas a reduction in inflammation during the tissue remodeling period in wound healing can reduce scar formation and improve skin wound healing outcomes [40–42]. Thus, the rapidly decreased inflammation reaction during the late stages of wound healing may serve as a protective factor, as demonstrated in this study. However, the mechanism mediating this special inflammation pattern in ECM/SVF-gel in vivo still remains unknown. Studies have revealed that platelets are exposed to collagen and other extracellular matrix components when blood components extravasate into the site of injury. This exposure causes the platelets not only to facilitate the formation of a hemostatic plug but also to secrete several mediators of wound healing, such as PDGF, that recruit neutrophils and macrophages to the wound site [43, 44]. Since ECM/SVF-gel contains concentrated ECM components, it may likely serve as a stimulus for the robust inflammation response during the early stages of wound healing. In cytotherapeutic strategies, MSCs have recently been shown to have various immunomodulatory effects on host immune cells in both wound healing and transplant biology [45–48]. To release immunosuppressive factors, MSCs can downregulate the inflammatory response and move the wound past the state of persistent inflammation, conferring the cells with immune privilege and making them an attractive cell type for the treatment of chronic wounds [49–51]. The density of ASCs in ECM/SVF-gel is significantly higher than that in Coleman fat and control fat [11], which may partly explain the rapid decline of inflammation in ECM/SVF-gel at the late stage of wound healing.

Adipocytes account for more than 90% of the volume of adipose tissue, whereas SVF cells and the native adipose ECM component account for only 10% of the volume [52]. In our study, preparation of ECM/SVF-gel equated to selectively eliminating mature adipocytes and maximizing enrichment of SVF cells and native adipose ECM. There is an abundance of evidence confirming the beneficial effects of native ECM on tissue-resident stem cells. In our study, SEM analysis revealed that ECM/SVF-gel contained a large amount of ECM components, and quantification in a previous study revealed that there were no significant differences in ECM integrity between ECM/SVF-gel and fresh adipose tissues [11]. Native ECM provides a physical bioscaffold for cellular support and retains the natural state [53–55], which may prevent rapid migration and unexpected stem cell behaviors [53, 56]. The ECM is known to serve as a natural reservoir for growth factors, and various potent angiogenic factors reside within the ECM scaffolds [57]. Moreover, the ECM is in a state of continuous remodeling catalyzed by matrix metalloproteinases in vivo [53, 58]. In vivo degradation of the ECM may release growth factors from the scaffold and produce degradation products (peptides), and these peptides have chemoattractant effects on endothelial cells both in vivo and in vitro [57]. These protective effects of the ECM component in ECM/SVF-gel may also contribute to the enhanced angiogenic response and final vascularization during the wound healing process. Besides providing structural support to the cells, the ECM also serves as a dynamic microenvironment that plays an important role in modulating resident cell behaviors and functions [59, 60]. Specifically, the elasticity [61, 62], nanotopography [63, 64], protein composition, and mechanical strain [65, 66] inherent to the ECM are all independent factors that regulate MSC function. Compared with the SVF suspension, the ECM scaffold in the ECM/SVF-gel accommodates ASCs within their natural niche, thereby providing a beneficial environment for attached ASCs to maintain optimal cell survival and potency.

Recent studies have described improvements in wound healing with autologous fat graft treatment, mainly depending on regenerative capacity and paracrine secretion ability provided by native ASCs [67–70]. Despite the wealth of evidence supporting their efficacy in wound healing, the volume occupied by adipocytes within the grafted fat significantly decreased the density of ASCs and the ECM, which may limit the therapeutic effects of the treatment. In this case, by enriching ASCs and the ECM, ECM/SVF-gel may maximize the therapeutic effect, offering an effective and safe option for wound healing.

## 5. Conclusion

In this study, we preliminarily verify the potential mechanisms underlying the efficacy of ECM/SVF-gel therapy on wound healing in a murine model. The results revealed that the therapeutic effect of ECM/SVF-gel is mainly attributed to the secretion of angiogenic factors and promotion of vascularization, and this process could be related to the intense inflammatory response in ECM/SVF-gel during the early stage of wound healing. Besides, the protective effects of the ECM component on attached ASCs in ECM/SVF-gel may also contribute to the enhanced angiogenic response and final wound healing process.
